# Draft Genome Sequence of *Brevibacillus brevis* DZQ7, a Plant Growth-Promoting Rhizobacterium with Broad-Spectrum Antimicrobial Activity

**DOI:** 10.1128/genomeA.00831-15

**Published:** 2015-08-20

**Authors:** Qihui Hou, Chengqiang Wang, Xiaoyang Hou, Zhilin Xia, Jiangping Ye, Kai Liu, Hu Liu, Jun Wang, Haimeng Guo, Xiaoning Yu, Yanan Yang, Binghai Du, Yanqin Ding

**Affiliations:** a College of Life Sciences, Shandong Agricultural University/Shandong Key Laboratory of Agricultural Microbiology, Taian, China; b Zunyi Tobacco Company, Zunyi, China

## Abstract

*Brevibacillus brevis* DZQ7 is a plant growth-promoting rhizobacterium (PGPR) isolated from tobacco rhizosphere. Here, we report the draft genome sequence of *B. brevis* DZQ7. Several functional genes related to antimicrobial activity were identified in the genome.

## GENOME ANNOUNCEMENT

*Brevibacillus brevis* (formerly *Bacillus brevis*), the type species of *Brevibacillus*, is considered a plant growth-promoting rhizobacterium (PGPR) (1–3) and organic pollutant degradation strain (4, 5). *B. brevis* is widespread in the soil and sediment, and it has been widely used in agriculture and environmental remediation because of its multiple potential functions (6, 7). *B. brevis* DZQ7 was isolated from the soil of tobacco rhizosphere in Guizhou, China. It showed broad-spectrum antimicrobial activity to pathogens of soilborne plant diseases, such as *Ralstonia solanacearum*, *Phytophthora nicotianae*, and *Fusarium* spp., and has been widely used in biological control of soilborne plant diseases.

Here, we report the draft genome sequence of *B. brevis* DZQ7. Genomic DNA was extracted and sequenced using the Illumina HiSeq 2500 platform. The whole-genome shotgun sequencing produced 1,243,319 paired-end reads with an average insert size of 500 bp (over 96-fold coverage). Another mate-paired (MP) library containing 5-kb inserts was constructed, and 5,041,325 paired-end reads (read length, 125 bp) were produced. The filtered reads were assembled by SOAPdenovo (8, 9) (http://soap.genomics.org.cn/soapdenovo.html) to generate scaffolds. All reads were used for further gap closure. Final assembly consisted of 3 scaffolds (length, ≥500 bp) with an *N*_50_ length of 6,158,809 bp, average length of 2,144,406 bp, and largest length of 6,158,809 bp. The genome sequence was annotated using the NCBI Prokaryotic Genome Automatic Annotation Pipeline (PGAAP) (http://www.ncbi.nlm.nih.gov/genome/annotation_prok/).

The genome consists of 6.43 Mb, with a G+C content of 47.42%, which is similar to those of *B. brevis* NBRC 100599 (47.3%) (NCBI reference sequence NC_012491), *B. brevis* FJAT-0809-GLX (47.3%) (GenBank accession no. AHKL00000000), and *B. brevis* X23 (46.8%) (GenBank accession no. AKYF00000000). A total of 5,762 coding sequences (CDS), 44 pseudogenes, 120 tRNA genes, and 29 rRNA genes were identified. As expected, many of the genes involved in the antimicrobial activity of *B. brevis* DZQ7 were identified in the genome, such as tyrocidine-synthetic gene, bacteriocin-synthetic gene, fusaricidin-synthetic gene, polyketide-synthetic (PKS) gene, chalcone-synthetic gene, nonribosomal peptide synthetic (NRPS) gene, and chitinase gene, and so on. In addition, *B. brevis* DZQ7 possesses genes with likely relevance to the degradation of organic pollutants, such as the phenylacetic acid degradation, phenylacetate degradation, and ethyl tert-butyl ether degradation genes.

The genome sequence of *B. brevis* DZQ7 and its curated annotation provides deeper insights into understanding the antimicrobial mechanism of *B. brevis*, which is critical for the development of a genetically engineered biocontrol agent. Also, it provides abundant genetic information regarding useful functions in bioremediation.

### Nucleotide sequence accession numbers.

The whole-genome shotgun project of *B. brevis* DZQ7 has been deposited in DDBJ/ENA/GenBank under the accession number LDZV00000000. The version described in this paper is the first version, LDZV00000000.1.

## References

[1] Jin F, Ding Y, Ding W, Reddy MS, Fernando WGD, Du B 2011 Genetic diversity and phylogeny of antagonistic bacteria against *Phytophthora nicotianae* isolated from tobacco rhizosphere. Int J Mol Sci 12:3055–3071. doi:10.3390/ijms12053055. 21686169 PMC3116175

[2] Che J, Liu B, Lin Y, Tang W, Tang J 2013 Draft genome sequence of biocontrol bacterium *Brevibacillus brevis* strain FJAT-0809-GLX. Genome Announc 1(2):e0016013. doi:10.1128/genomeA.00160-13. 23618715 PMC3636543

[3] Chen W, Wang Y, Li D, Li L, Xiao Q, Zhou Q 2012 Draft genome sequence of *Brevibacillus brevis* strain x23, a biocontrol agent against bacterial wilt. J Bacteriol 194:6634–6635. doi:10.1128/JB.01312-12. 23144389 PMC3497470

[4] Ye J, Zhao H, Yin H, Peng H, Tang L, Gao J, Ma Y 2014 Triphenyltin biodegradation and intracellular material release by *Brevibacillus brevis*. Chemosphere 105:62–67. doi:10.1016/j.chemosphere.2013.12.039. 24388446

[5] Liao L, Chen S, Peng H, Yin H, Ye J, Liu Z, Dang Z, Liu Z 2015 Biosorption and biodegradation of pyrene by *Brevibacillus brevis* and cellular responses to pyrene treatment. Ecotoxicol Environ Saf 115:166–173. doi:10.1016/j.ecoenv.2015.02.015. 25700095

[6] Yang JH, Liu HX, Zhu GM, Pan YL, Xu LP, Guo JH 2008 Diversity analysis of antagonists from rice-associated bacteria and their application in biocontrol of rice diseases. J Appl Microbiol 104:91–104. doi:10.1111/j.1365-2672.2007.03534.x. 17850318

[7] Tang S, Bai J, Yin H, Ye J, Peng H, Liu Z, Dang Z 2014 Tea saponin enhanced biodegradation of decabromodiphenyl ether by *Brevibacillus brevis*. Chemosphere 114:255–261. doi:10.1016/j.chemosphere.2014.05.009. 25113210

[8] Li R, Zhu H, Ruan J, Qian W, Fang X, Shi Z, Li Y, Li S, Shan G, Kristiansen K, Li S, Yang H, Wang J, Wang J 2010 *De novo* assembly of human genomes with massively parallel short read sequencing. Genome Res 20:265–272. doi:10.1101/gr.097261.109. 20019144 PMC2813482

[9] Li R, Li Y, Kristiansen K, Wang J 2008 SOAP: short oligonucleotide alignment program. Bioinformatics 24:713–714. doi:10.1093/bioinformatics/btn025. 18227114

